# Assessment of Variation in Electronic Health Record Capabilities and Reported Clinical Quality Performance in Ambulatory Care Clinics, 2014-2017

**DOI:** 10.1001/jamanetworkopen.2021.7476

**Published:** 2021-04-22

**Authors:** Paul G. Shekelle, Joseph D. Pane, Denis Agniel, Yunfeng Shi, Juliet Rumball-Smith, Ann Haas, Shira Fischer, Robert S. Rudin, Mark Totten, Julie Lai, Dennis Scanlon, Cheryl L. Damberg

**Affiliations:** 1 Department of Health Care, RAND Corporation, Santa Monica, California; 2 West Los Angeles Veterans Administration, Los Angeles, California; 3 Department of Economics, Sociology, and Statistics, RAND Corporation, Pittsburgh, Pennsylvania; 4 Department of Health Policy and Administration, Pennsylvania State University, University Park; 5 Ministry of Health, Wellington, New Zealand; 6 Precision Driven Health, Auckland, New Zealand; 7 Department of Health Care, RAND Corporation, Pittsburgh, Pennsylvania; 8 Department of Health Care, RAND Corporation, Boston, Massachusetts; 9 Department of Research Programming, RAND Corporation, Santa Monica, California

## Abstract

**Importance:**

Electronic health records (EHRs) are widely promoted to improve the quality of health care, but information about the association of multifunctional EHRs with broad measures of quality in ambulatory settings is scarce.

**Objective:**

To assess the association between EHRs with different degrees of capabilities and publicly reported ambulatory quality measures in at least 3 clinical domains of care.

**Design, Setting, and Participants:**

This cross-sectional and longitudinal study was conducted using survey responses from 1141 ambulatory clinics in Minnesota, Washington, and Wisconsin affiliated with a health system that responded to the Healthcare Information and Management Systems Society Annual Survey and reported performance measures in 2014 to 2017. Statistical analysis was performed from July 10, 2019, through February 26, 2021.

**Main Outcomes and Measures:**

A composite measure of EHR capability that considered 50 EHR capabilities in 7 functional domains, grouped into the following ordered categories: no functional EHR, EHR underuser, EHR, neither underuser or superuser, EHR superuser; as well as a standardized composite of ambulatory clinical performance measures that included 3 to 25 individual measures (median, 13 individual measures).

**Results:**

In 2014, 381 of 746 clinics (51%) were EHR superusers; this proportion increased in each subsequent year (457 of 846 clinics [54%] in 2015, 510 of 881 clinics [58%] in 2016, and 566 of 932 clinics [61%] in 2017). In each cross-sectional analysis year, EHR superusers had better clinical quality performance than other clinics (adjusted difference in score: 0.39 [95% CI, 0.12-0.65] in 2014; 0.29 [95% CI, −0.01 to 0.59] in 2015; 0.26 [95% CI, –0.05 to 0.56] in 2016; and 0.20 [95% CI, –0.04 to 0.45] in 2017). This difference in scores translates into an approximately 9% difference in a clinic’s rank order in clinical quality. In longitudinal analyses, clinics that progressed to EHR superuser status had only slightly better gains in clinical quality between 2014 and 2017 compared with the gains in clinical quality of clinics that were static in terms of their EHR status (0.10 [95% CI, −0.13 to 0.32]). In an exploratory analysis, different types of EHR capability progressions had different degrees of associated improvements in ambulatory clinical quality (eg, progression from no functional EHR to a status short of superuser, 0.06 [95% CI, −0.40 to 0.52]; progression from EHR underuser to EHR superuser, 0.18 [95% CI, −0.14 to 0.50]).

**Conclusions and Relevance:**

Between 2014 and 2017, ambulatory clinics in Minnesota, Washington, and Wisconsin with EHRs having greater capabilities had better composite measures of clinical quality than other clinics, but clinics that gained EHR capabilities during this time had smaller increases in clinical quality that were not statistically significant.

## Key Points

**Question:**

Is the adoption of more advanced electronic health record (EHR) capabilities associated with better ambulatory clinical quality of care?

**Findings:**

In cross-sectional analyses of 3 states from 2014 to 2017, ambulatory clinics (769-972 per year) with more advanced EHR capabilities had higher scores on a composite measure of ambulatory clinical quality than did other clinics, which translated into an approximately 9% difference in rank order of quality. Across the much smaller number of clinics that gained advanced EHR capabilities between 2014 and 2017 (n = 72), the composite of ambulatory clinical quality improved more than for other clinics, but the difference was not statistically significant.

**Meaning:**

This study suggests that ambulatory clinics with advanced EHR capabilities were associated with a better performance on a composite measure of ambulatory clinical quality than clinics with less-advanced EHR capabilities; clinics that adopted advanced EHR capabilities during a 3-year period were not associated with significant increases in ambulatory clinical quality performance.

## Teaser

This cross-sectional study assesses the association between electronic health records with different degrees of capabilities and publicly reported ambulatory quality measures in at least 3 clinical domains of care.

## Introduction

Patients, payors, and clinicians all desire high-quality health care, yet the goal remains elusive. In 2003, McGlynn and colleagues^1^ documented substantial shortcomings in the quality of care, and an updated analysis of ambulatory quality through 2013 continued to show large gaps in care.^2^ More recently, an assessment of medical groups in Minnesota found that none were able to achieve 90% or greater performance on a small number of common publicly reported ambulatory quality measures.^3^ A low threshold of 60% was needed before any medical groups could be identified that met this standard of care across all measures. The gap between the aspiration of high-quality care and actual care delivery remains wide.

For more than 15 years, the electronic health record (EHR) has been considered to be an important tool in the quest for high-quality care, based on the following model: the data storage and analytic capabilities of the EHR vastly exceed noncomputerized alternatives, which then enable and allow for some of the tasks and tools believed necessary to improve quality, such as clinical registries and decision support. Systematic reviews of dozens of hypothesis-testing studies, in general, support this belief, although these studies are mostly about specific EHR capabilities and their associated outcome, and many were conducted in academic health center hospitals. For example, in 1 broad-based systematic review, the most commonly identified study type was of clinical decision support targeted at a specific process or outcome, such as improving cancer screening or medication management.^4^ Studies of EHR use in ambulatory care have been limited by 1 or more of the following: use of a cross-sectional design (meaning causality cannot be inferred), the classification of the EHR as a binary yes or no variable (which does not distinguish between EHRs with basic functionalities and those with many capabilities), or having a narrow focus in terms of patients (eg, patients with diabetes or HIV), providers (studies of a single institution or provider system), or outcomes (eg, preventive screening or immunizations).^5,6,7,8,9,10,11,12,13,14,15,16,17^ The hypothesis has yet to be tested that implementation of an advanced EHR with many capabilities is associated with broad-based improvements in the quality of ambulatory care across a large spectrum of community-based providers.

To assess this hypothesis, we used data from the Healthcare Information and Management Systems Society (HIMSS) Annual Survey for the years 2014 to 2017 and publicly reported clinical quality measures in 3 states to assess the association between increasing EHR capabilities and differences in clinical quality performance in multiple domains of ambulatory performance, using both cross-sectional and longitudinal samples.

## Methods

This study was performed as part of the RAND Center of Excellence on Health System Performance, funded through a cooperative agreement with the Agency for Healthcare Research and Quality. The RAND Center of Excellence on Health System Performance partnered with 4 states that publicly report quality measures: California, Minnesota, Washington, and Wisconsin. California reports performance at the physician organization level and, therefore, is not included in this analysis, which is at the ambulatory clinic level. We examine both cross-sectional and longitudinal measures of EHR capability and performance. This analysis received a waiver of written consent and expedited review from the RAND institutional review board. This study followed the Strengthening the Reporting of Observational Studies in Epidemiology (STROBE) reporting guideline.

### EHR Capabilities

The data come from annual surveys of ambulatory clinics, provided by the HIMSS Analytics LOGIC Market Intelligence Platform. The HIMSS surveys collected detailed information on the capabilities of health information technology for more than 75% of ambulatory care clinics affiliated with health systems. The HIMSS defines a health system as an organization that owns at least 1 hospital. A previous study has shown agreement between HIMSS survey data and similar data collected by Minnesota Community Measurement on Minnesota clinics to be sufficiently good to have confidence in the results at the level of the presence or absence of major EHR functionalities, such as computerized clinician order entry, medication-based decision support, and e-prescribing.^18^ For the cross-sectional analysis, we included ambulatory clinics that responded to the survey and had clinical quality measures reported for that year. For the longitudinal analysis, we included ambulatory clinics that responded to the survey and had clinical quality measures reported in 2014 and 2017. The HIMSS collects data on approximately 50 functionalities, almost all of which are about capabilities such as “basic medication screening (drug-drug, drug-allergy),” “all lab reports are electronically imported and stored in discrete structured form,” and “a patient portal allowing the patient to see personal health information, pay bills, request a schedule, request an appointment, etc,” in 7 general domains of EHR capability such as data repository, clinical decision support, and order entry management; 1 data point is about use (use of e-prescribing for ≥75% of orders) (eTable 1 in the Supplement). We used the method developed by Rumball-Smith et al,^19^ which sums the number of functions within a domain and then the number of domains within the EHR to aggregate these at the clinic level into the categories of EHR underuser, superuser, or neither superuser nor underuser (a middle category), or no functional EHR. Thus, the progression of increasing EHR capabilities goes from no functional EHR (which is self-explanatory) to EHR underuser (eg, an EHR that is primarily electronic capture of clinical data, with perhaps registry capabilities) to EHR user, neither superuser or underuser (eg, adding e-prescribing and basic clinical decision support), to EHR superuser (eg, adding advanced clinical decision support, a patient portal, and electronic health information exchange).

### Clinical Quality Performance

We used data that are publicly posted online about clinical quality performance for Minnesota,^20^ Washington,^21^ and Wisconsin.^22^ Each state reported a different set of standard clinical quality measures (eTable 2 in the Supplement lists all measures for each state that were available for use). For this analysis, we included only clinics that reported quality measures in at least 3 domains of care (eg, cancer screening, diabetes, and vascular disease or asthma, medication safety, and depression) because we were looking for broad-based differences in quality rather than a focus on 1 or 2 health conditions. We used the method of Agniel et al^23^ to construct a composite measure of clinical quality performance. In brief, this method aggregates multiple measures of performance using a 1-parameter Rasch model that is often used in item-response theory and psychological research. The method models the probability of passing a given measure as a function of 2 aspects: the difficulty of the measure and the overall composite quality of the clinician. The difficulty of the measure is defined as the log odds of passing the measure for an average clinician. In other words, clinicians get more credit in the composite for performing better on measures that other clinicians find hard to meet than they do for performing better on measures that almost all clinicians meet easily. Composite measures for each clinic in each year were obtained as mean a posteriori estimates from a bayesian model. Models were fit on each state separately, and composites in each state were normalized to a scale with mean zero and unit SD. Analyses were restricted within states to measures that were sufficiently unchanged during the time period. Based on these estimates, we limited our analysis to clinics whose composite was estimated with a reliability of at least 0.9, where reliability was computed as the ratio of the variance of the composite to the total variance.

### Matching Clinics Across Data Sets

With names, addresses, and telephone numbers, we matched the clinics in the outcome data set to the clinics in the HIMSS surveys using SAS, version 9.4 (SAS Institute Inc).^24^ We then manually validated all the matched clinics. A similar approach was used in a previous study that also used HIMSS data.^18^

### Statistical Analysis

Statistical analysis was performed from July 10, 2019, through February 26, 2021. We used these matched statewide data sets to conduct 2 analyses, each with strengths and limitations. One analysis was cross-sectional, which has the advantage of a much greater sample size but is limited by its inability to establish a temporal association between EHR capabilities and clinical quality performance. For this analysis, we compared the composite measure of clinical quality between clinics categorized either as having no functional EHR or as EHR underusers with clinics categorized either as EHR superusers or as EHR users, neither superuser nor underuser. The dependent variable was the standardized composite (standardized to have the same variance in each state) for the appropriate year, and the only independent variable was that year’s EHR capability. For ease of presentation, we then combined the non-superuser categories into 1 category (owing to very small sample sizes for the category of no functional EHR and the similar estimates for the other 2 categories). Our second analysis was longitudinal, assessing change over time. This analysis captures the temporality of the EHR-performance association—we can compare the quality performance of clinics before and after changes in EHR capabilities—but is necessarily more limited in sample size because the included clinics need to report both EHR data and performance data across time, and only a limited number of clinics change their EHR capabilities over time. These longitudinal analyses required that an ambulatory clinic reported performance data in 2014 and 2017. For the comparison group, we used the clinics whose EHR status was stable from 2014 to 2017 at one of the following statuses: no functional EHR, EHR underuser, or EHR user, neither underuser or superuser. We compared clinics that progressed to superuser status or clinics that progressed in their EHR functionalities but not all the way to superuser status (such as from no functional EHR to EHR underuser) with the comparison group. We performed 2 longitudinal comparisons. Each comparison tested a slightly different construct. The dependent variable in the first longitudinal comparison was the difference in the standardized composite from 2014 to 2017. In the second comparison, the dependent variable was the standardized composite in 2017. In all analyses, we performed a linear regression model adjusting for state with clustering of clinics at the health system level. All analyses were adjusted for 3 variables available in the HIMSS database: whether an ambulatory care site was a primary care clinic, the number of physicians, and whether the ambulatory care site belonged to a single hospital or multihospital system. We used a cluster-robust variance estimation for small sample methods.^25^ All analyses were conducted using R, version 3.6.0 (R Group for Statistical Consulting). All *P* values were from 2-sided tests and results were deemed statistically significant at *P* < .05.

### Placing Composite Scores in Context

We used linear regression models to quantify the following questions: if 2 clinics differ by 1 unit on the composite, how much, on average, do they differ in terms of (1) the component measures, (2) rank on the component measures, and (3) rank on the composite measure? Estimates for these differences were calculated by regressing the corresponding quantities on the composite in each year and (for measures 1 and 2) for each measure. To simplify presentation, regression estimates were averaged across years.

## Results

Across all 3 states and years, there were 3752 ambulatory care clinics in the HIMSS database and 2915 clinics that had publicly reported quality data. We were able to match 46% of the ambulatory clinic sites (1707 of 3752) to responses in the HIMSS database, which captured 68% of the clinics (1968 of 2915) that had publicly reported quality data. A primary reason for clinics failing to match responses in the HIMSS database was that the HIMSS reports data only for clinics that are part of health systems, and many clinics are not part of systems. After filtering clinics with fewer than 3 clinical domains of care or a composite measure with insufficient reliability, we were left with 1141 unique ambulatory care sites, 30% of the original HIMSS sample (eFigures 1-5 in the Supplement), matching with 1192 clinics (41%) that had publicly reported quality data. Of the matched clinics, in 2014, 381 of 746 clinics (51%) were already EHR superusers. By 2017, this number had increased to 566 of 932 clinics (61%). Table 1 presents the descriptive data about these clinics from the HIMSS database. Primary care clinics comprised 55% (209 of 377) to 63% (128 of 204) of the sample, and most clinics were part of multihospital health systems. Clinics had a median of 5 to 6 physicians per clinic and reported a median of 6 measure domains in Minnesota (range, 3-6 measure domains), 10 measure domains in Washington (range, 3-12 measure domains), and 7 measure domains in Wisconsin (range, 3-7 measure domains). Across all 3 states, the median number of measure domains included in the clinical quality composite was 6 (interquartile range, 3-12 measure domains). The clinical composite scores for clinics that did not match with HIMSS data were, in general, somewhat smaller than those that did match, but except for 2014, differences were small (eTable 3 in the Supplement). After comparison of clinics that had HIMSS data but did not match to a report on clinical quality, it was found that, in general, unmatched clinics were in systems that had a smaller percentage of primary care clinics, a smaller number of clinics in multihospital systems, and a smaller number of physicians per clinic (eTable 4 in the Supplement).

**Table 1.** Characteristics of Ambulatory Care Clinics in Cross-Sectional Analysis

State and year	No. of clinics	No. of health systems	No. of clinics per health system, median (IQR)	No. (%)		No. of clinics reporting No. of physicians in clinic	No. of physicians per clinic, median (IQR)
				Primary care clinics	Clinics in multihospital systems		
Minnesota							
2014	379	46	2 (1-6)	208 (55)	316 (83)	374	6 (2-12)
2015	358	42	2 (1-7)	201 (56)	293 (82)	355	6 (2-12)
2016	417	43	4 (1-9)	233 (56)	334 (80)	409	6 (2-11)
2017	377	41	3 (1-7)	209 (55)	304 (81)	373	6 (2-11)
Washington							
2014	186	26	3 (2-12)	115 (62)	120 (65)	175	6 (3-10)
2015	248	42	2 (1-7)	144 (58)	177 (71)	239	5 (3-9)
2016	245	39	2 (1-6)	147 (60)	181 (74)	229	5 (3-9)
2017	341	46	1.5 (1-7)	161 (47)	258 (76)	316	5 (2-9)
Wisconsin							
2014	204	18	10.5 (4-18)	128 (63)	169 (83)	197	5 (3-9)
2015	264	19	13 (7-19)	160 (61)	236 (89)	252	6 (3-11)
2016	254	18	11.5 (4-19)	154 (61)	226 (89)	243	5 (3-11)
2017	254	17	13 (6-19)	155 (61)	248 (98)	243	6 (3-11)

Abbreviation: IQR, interquartile range.

### Cross-Sectional Results

Table 2 shows the results of our cross-sectional analysis. Between 2014 and 2017, the number of clinics classified as EHR superusers increased from 381 to 566. In each year, clinics that were classified as superusers had better clinical quality composite scores than clinics that were not EHR superusers (adjusted difference in score: 0.39 [95% CI, 0.12-0.65] in 2014; 0.29 [95% CI, −0.01 to 0.59] in 2015; 0.26 [95% CI, –0.05 to 0.56] in 2016; and 0.20 [95% CI, –0.04 to 0.45] in 2017). This difference in scores translates into an approximately 9% difference in a clinic’s rank order in clinical quality. A 0.20 difference in the clinical quality composite measure means that clinics scoring better would have, for example, a 1.8, 3.0, and 3.7 absolute percentage point better score on colorectal cancer screening in Washington, Wisconsin, or Minnesota, respectively (because the clinical composites are estimated from different measures and standardized within the state, the meaning of a 0.20 difference varies across states). For performance measures of diabetes testing or control, the respective differences would be a 0.7, 2.5, and 2.7 absolute percentage point gain in Washington, Wisconsin, and Minnesota. These differences equate to a change in position of 18 to 51 places on a rank order of clinics’ performance, depending on performance measure and state.

**Table 2.** Cross-Sectional Analysis of Composite Clinical Quality Between Clinics With EHR Superuser Status and Other Clinics

Year	Clinics, No.		Clinical quality composite, superuser compared with all others (95% CI)^a^	*P* value
	Superuser	All others		
2014	381	365	0.39 (0.12 to 0.65)	.01
2015	457	389	0.29 (−0.01 to 0.59)	.06
2016	510	371	0.26 (−0.05 to 0.56)	.09
2017	566	366	0.20 (−0.04 to 0.45)	.10

Abbreviation: EHR, electronic health record.

^a^ Adjusted for whether a clinic is a primary care clinic, the number of physicians, and whether the clinic is part of a single-hospital system.

### Longitudinal Results

Table 3 shows the results of our longitudinal analysis. Between 2014 and 2017, 72 clinics progressed to EHR superuser status, and another 33 clinics progressed from their 2014 status to something less than superuser status. A total of 519 clinics were static, of which 339 were EHR superusers and 180 clinics were something else (no functional EHR, underusers, or neither superuser nor underuser) (eTable 5 in the Supplement). The relative standing of the 339 static superusers declined from 2014 to 2017 compared with their peers because their standardized clinical quality composite scores were, on average, increasing less quickly than non-superuser clinics (−0.18 [95% CI, −0.33 to −0.04]), although their overall 2017 quality remained high compared with clinics that were static with respect to other categories of EHR capability (0.29 [95% CI, 0.03-0.56]). In 2017, clinics that progressed in their EHR capabilities to superuser status had a relatively small increase in clinical quality (0.10 [95% CI, −0.13 to 0.32]), although their overall quality was higher than clinics that remained static (0.21 [95% CI, −0.03 to 0.44]).

**Table 3.** Longitudinal Analysis of Progression in EHR Capability and Clinical Quality Composite

EHR classification over time	Clinical quality composite, superuser compared with all static non-superuser clinic (95% CI)^a^	*P* value
Outcome is the difference between 2017 and 2014 clinical quality composite		
Intercept	0.15 (−0.03 to 0.33)	.10
Static superuser clinics	−0.18 (−0.33 to −0.04)	.02
Clinics that progressed short of superuser status	0.03 (−0.24 to 0.30)	.82
Clinics that progressed to superuser status	0.10 (−0.13 to 0.32)	.36
Outcome is the 2017 clinical quality composite		
Intercept	0.00 (−0.23 to 0.22)	.98
Static superuser clinics	0.29 (0.03 to 0.56)	.03
Clinics that progressed short of superuser status	0.09 (−0.24 to 0.41)	.56
Clinics that progressed to superuser status	0.21 (−0.03 to 0.44)	.08

Abbreviation: EHR, electronic health record.

^a^ Adjusted for whether a clinic is a primary care clinic, the number of physicians, and whether the clinic is part of a single-hospital system.

In an exploratory analysis (Table 4), this overall value was qualitatively different depending on the type of EHR transition that occurred, with small changes for the transitions of not functional EHR to either underuser or neither (0.06 [95% CI, −0.40 to 0.52]), from underuser to neither (–0.03 [95% CI, −0.37 to 0.31]), and from neither to superuser (0.05 [95% CI, −0.30 to 0.41]) and a larger change for clinics transitioning from underuser to superuser (0.18 [95% CI, −0.14 to 0.50]), although the 95% CIs overlapped for all groups. The 105 clinics that progressed in EHR capabilities were part of 20 different health systems, including large health systems, such as Sanford Health and Virginia Mason (each with 18 clinics that progressed in EHR capability), and smaller health systems, such as Overlake Medical Center and Clinics (with 3 progressing clinics).

**Table 4.** Longitudinal Analysis of Specific Progressions in EHR Capability and Clinical Quality Composite

EHR classification over time	Clinical quality composite compared with all other static clinics (95% CI)^a^	*P* value
Outcome is the difference between 2017 and 2014 clinical quality composite		
Intercept	0.15 (−0.04 to 0.33)	.11
Static superuser clinics	−0.18 (−0.33 to −0.04)	.02
Clinics that progressed from		
Underusers to neither	−0.03 (−0.37 to 0.31)	.78
Not functional EHR to underusers or neither	0.06 (−0.40 to 0.52)	.73
Neither to superusers	0.05 (−0.30 to 0.41)	.71
Underusers to superusers	0.18 (−0.14 to 0.50)	.17

Abbreviation: EHR, electronic health record.

^a^ Adjusted for whether a clinic is a primary care clinic, the number of physicians, and whether the clinic is part of a single-hospital system.

## Discussion

One principal finding from this analysis is that EHR superuser clinics consistently had, on average, better clinical quality performance than non-superuser clinics, but these results are compatible with both larger effects and essentially null effects. Although the absolute difference on any individual performance measure may have been small, these small improvements are aggregated across a median of 13 measures. The net difference (as measured by the clinical composite) may be more clinically important. A change of 0.20 in the clinical composite measure, which is at the lower end of the range of observed results for the cross-sectional analyses, is equivalent to a change in rank order of about 45 to 75 places if starting near the top end of the rank order (80th percentile) or 85 to 95 places if starting in the middle of the rank order (with the total number of clinics, in general, between 600 and 1200, depending on state).

A second principal finding is that, over time, all clinics, on average, had increased clinical quality; clinics that transitioned to EHR superuser status had larger increases in quality than did clinics with static EHR user status. However, this difference in superuser clinic quality was not as big as the differences between superuser and non-superuser clinics in the cross-sectional analysis, and it was not statistically significant.

What can account for the differences in results between the cross-sectional analysis and the longitudinal analysis? One explanation is that multifunctional EHR systems add nothing to clinical quality improvement, that, in the cross-sectional analysis, the clinics with multifunctional EHRs were good-quality clinics to begin with, and that the EHR was not associated with their higher clinical quality score. Although possible, this explanation is at variance with the conclusions from systematic reviews and meta-analyses of hypothesis-testing studies of individual EHR capabilities in narrower settings and/or with a narrower focus.^4,26^ Another possible explanation is that it takes more than 3 years to realize gains in clinical quality from expanded EHR capabilities. This explanation would be compatible with the notion that EHR implementation is a sociotechnical intervention, and after adding EHR capabilities, a period of time is required for the changes in workflow necessary to optimize results.^27,28^ A third possible explanation is that the gains in clinical quality owing to expanded EHR capabilities are not large enough to be detected with these sample sizes in a situation in which the baseline change in clinical quality is, on average, moderately large. A fourth explanation, suggested by our exploratory analysis, is that not all EHR capability transitions are the same; that over a 2- to 3-year period, the “sweet spot” is a clinic that already has an EHR but is underusing its capabilities; and that adding enough capabilities to achieve superuser status is associated with clinical quality increases of about the same magnitude as seen in our cross-sectional analysis. These are all hypotheses for future studies, which have implications for how best to achieve clinical quality increases in the short and medium term.

### Limitations

This analysis has several limitations. The EHR capabilities come from a survey completed by HIMSS personnel based on an interview with each ambulatory clinic and are subject to the limitations any such survey has that what is recorded as being present or absent is a representation and not a direct measurement. However, we compared the results of the HIMSS survey in Minnesota with an independent survey of EHR capabilities of Minnesota ambulatory clinics and found good agreement on the presence or absence of major capabilities.^18^ We also used a composite of EHR capabilities as our estimator variable, rather than try to assess associations between highly specific capabilities (such as a clinical reminder for colorectal cancer screening) and a specific performance measure (colorectal cancer screening). This choice was deliberate because we were interested in the multifunctionality of the EHR, which may be associated with care through myriad pathways (eg, effective blood pressure control may be associated with the use of data repository capabilities to create clinical registries, decision support for a clinical reminder, secure messaging between clinician and patient, health information exchange, and possibly other ways). The HIMSS data are also restricted to ambulatory clinics affiliated with a health system; the EHR data about unaffiliated clinics have only recently been released and are still incomplete. Although this limitation may affect the generalizability of our findings to clinics outside of health systems, it should not affect the internal validity. As health systems continue to expand, ambulatory clinics affiliated with health systems will become the dominant type of ambulatory clinic in the US. In addition, the 14 or 21 or 25 measures included in the publicly reported data sets in the 3 states do not constitute a complete picture of ambulatory clinical quality. Nevertheless, they do cover many important dimensions of care with high prevalence in the population and are intended to be used by patients and clinicians to identify higher-performing clinicians, and thus they seem acceptable to use for our purpose of assessing the association of EHRs with clinical quality. We used these measures to create a composite rather than to assess each one individually. This was done for practical and theoretical reasons (clinics reported different sets of measures, and we were interested in broad measures of EHR capabilities and broad measures of quality), but we followed, to the extent possible, suggestions for composites regarding transparency, technical reproducibility, and statistical fitness.^29^ Another limitation is that we were able to match only between 43% and 75% of eligible clinics from the health partner data to an ambulatory care site from HIMSS and only between 17% and 44% of the ambulatory care sites in HIMSS to a clinic in the health partner data. The clinics that did not match had clinical quality composite scores that were roughly comparable to those of the clinics that did match, but unmatched clinics were in systems that were smaller and less focused on primary care. This matching challenge is also more a limitation of the generalizability than the internal validity of our results.

## Conclusions

We found that, between 2014 and 2017, ambulatory clinics in Minnesota, Washington, and Wisconsin that had EHRs with greater capabilities had better clinical quality than other clinics, but clinics that gained EHR capabilities over time had smaller, and not statistically significant, increases in clinical quality over time. These results are consistent with several hypotheses, including that increasing EHR capability is associated with no or, at best, modest improvements in clinical quality; improvements in clinical quality associated with increasing EHR capabilities take several years to be realized. Over 2 to 3 years, the largest clinical quality increase was in clinics that already have a functioning EHR that is being underused in terms or its capabilities. As US health care continues to evolve and clinicians gain more EHR capabilities, our results and future studies of the new hypotheses generated will be vital to efforts to improve ambulatory clinical quality.

## Notes

Supplement. **eTable 1.** Electronic Health Record Capabilities Assessed by HIMSS **eTable 2.** Performance Measures Publicly Reported at the Clinic Level in 3 States **eTable 3.** Clinical Quality Composite Scores of Matched and Unmatched Ambulatory Care Sites in Cross-Sectional Analysis **eTable 4.** Characteristics of Matched and Unmatched Ambulatory Care Sites in Cross-Sectional Analysis **eTable 5.** Longitudinal Analysis: Clinic Capabilities **eFigure 1.** Matching of Clinics in Washington, Wisconsin, and Minnesota, 2014 Cross-Sectional Analysis **eFigure 2.** Matching of Clinics in Washington, Wisconsin, and Minnesota, 2015 Cross-Sectional Analysis **eFigure 3.** Matching of Clinics in Washington, Wisconsin, and Minnesota, 2016 Cross-Sectional Analysis **eFigure 4.** Matching of Clinics in Washington, Wisconsin, and Minnesota, 2017 Cross-Sectional Analysis **eFigure 5.** Matching of Clinics in Washington, Wisconsin, and Minnesota, Longitudinal Analysis, 2014-2017
