# Supplementary material for: Assessment of Variation in Electronic Health Record Capabilities and Reported Clinical Quality Performance in Ambulatory Care Clinics, 2014-2017
Source: JAMA Netw Open. 2021 Apr 22;4(4):e217476. doi: 10.1001/jamanetworkopen.2021.7476 (PMC8063064; doi:10.1001/jamanetworkopen.2021.7476)
Supplement: Supplement. — eTable 1. Electronic Health Record Capabilities Assessed by HIMSS eTable 2. Performance Measures Publicly Reported at the Clinic Level in 3 States eTable 3. Clinical Quality Composite Scores of Matched and Unmatched Ambulatory Care Sites in Cross-Sectional Analysis eTable 4. Characteristics of Matched and Unmatched Ambulatory Care Sites in Cross-Sectional Analysis eTable 5. Longitudinal Analysis: Clinic Capabilities eFigure 1. Matching of Clinics in Washington, Wisconsin, and Minnesota, 2014 Cross-Sectional Analysis eFigure 2. Matching of Clinics in Washington, Wisconsin, and Minnesota, 2015 Cross-Sectional Analysis eFigure 3. Matching of Clinics in Washington, Wisconsin, and Minnesota, 2016 Cross-Sectional Analysis eFigure 4. Matching of Clinics in Washington, Wisconsin, and Minnesota, 2017 Cross-Sectional Analysis eFigure 5. Matching of Clinics in Washington, Wisconsin, and Minnesota, Longitudinal Analysis, 2014-2017 [file jamanetwopen-e217476-s001.pdf]

Supplementary Online Content

Shekelle PG, Pane JD, Agniel D, et al. Assessment of variation in electronic health record capabilities and reported clinical quality performance in ambulatory care clinics, 2014-2017. *JAMA Netw Open.* 2021;4(4):e217476.
doi:10.1001/jamanetworkopen.2021.7476

eTable 1. Electronic Health Record Capabilities Assessed by HIMSS
eTable 2. Performance Measures Publicly Reported at the Clinic Level in 3 States
eTable 3. Clinical Quality Composite Scores of Matched and Unmatched Ambulatory Care Sites in Cross-Sectional Analysis
eTable 4. Characteristics of Matched and Unmatched Ambulatory Care Sites in Cross-Sectional Analysis
eTable 5. Longitudinal Analysis: Clinic Capabilities
eFigure 1. Matching of Clinics in Washington, Wisconsin, and Minnesota, 2014 Cross-Sectional Analysis
eFigure 2. Matching of Clinics in Washington, Wisconsin, and Minnesota, 2015 Cross-Sectional Analysis
eFigure 3. Matching of Clinics in Washington, Wisconsin, and Minnesota, 2016 Cross-Sectional Analysis
eFigure 4. Matching of Clinics in Washington, Wisconsin, and Minnesota, 2017 Cross-Sectional Analysis
eFigure 5. Matching of Clinics in Washington, Wisconsin, and Minnesota, Longitudinal Analysis, 2014-2017

This supplementary material has been provided by the authors to give readers additional information about their work.

© 2021 Shekelle PG et al. *JAMA Network Open.*

eTable 1. Electronic Health Record Capabilities Assessed by HIMSS

**Domain: Use of data repository capacity**	Clinical research data analysis
	Current encounter procedures
	Current encounter vital signs including height, weight, blood pressure, temperature, etc.
	Nursing documentation
	Physician documentation
	Problem lists
	Structured document templates (e.g. diabetic workup, annual physical, etc.) creating discrete data
	Transcribed reports are stored electronically
	Ability to create growth charts from the capture of structured data (vital signs, immunizations, BMI, etc.)
	Ability to incorporate current encounter procedures into standardized format (e.g. CCD, CCR)
	Medication lists on-line for all patients
	Medication reconciliation
	Problem lists
	Structured document templates (e.g. diabetic workup, annual physical, etc.) creating discrete data
	Transcribed reports are stored electronically
	Ability to create growth charts from the capture of structured data (vital signs, immunizations, BMI, etc.)
	Ability to incorporate current encounter procedures into standardized format (e.g. CCD, CCR)
	Medication lists on-line for all patients
	Medication reconciliation
**Domain: Clinical decision support**	Basic medication screening (drug/drug, drug/allergy)
	Clinical guidelines or protocols

© 2021 Shekelle PG et al. *JAMA Network Open.*

	Data from the community based EHR is incorporated into the EMR's rules engine and triggers alerts
	Genomics profiling is incorporated into the EMR and could result in a suggested order or order change
	Preventive medicine (e.g. immunizations, follow-up testing)
	Receipt of diagnostics results trigger relevant clinical alerts and clinical guidance/recommended care
	Remote device monitoring process alerts clinician when clinically significant changes in data are detected
	Capable of comparing patient follow-up recommendations to care rendered by all providers with access to the community-based EMR and variance and compliance alerts are generated
	EMR suggests recommended follow-up based on date, patient problem list and procedures rendered by current provider and others.
	Follow-up notices sent to the patients are initiated by flags set by provider
**Domain: Order entry management**	Ability to find and modify orders for all patients on a specific medication
	e-Prescribing for new medications
	e-Prescribing for refill medication requests
	75% or more orders completed in this way
**Domain: Electronic messaging**	Consult communications
	Disease management communications
	Internal clinic communications
	Patient communications
	Referral communications
**Domain: Results management**	All lab reports are electronically imported and stored in discrete structured form OR Textual/data results may be returned via HL 7 transactions and stored directly into patient records
	Textual/data results returned electronically in formats such as PDF, CCR, and CCD, and then attached to patient record

© 2021 Shekelle PG et al. *JAMA Network Open.*

	Output from diagnostic and intelligent medical devices are incorporated directly into patient's EMR when appropriate.
**Domain: Health information exchange**	Capable of exchanging data across multiple vendor platforms for the purpose of health information exchange
	Web browser on physician/nurse desktops for access to online reference material, eligibility information, lab results, etc.
	With external registries for reporting of patient data (e.g. immunization, disease or device)
	With governmental agencies (e.g. local, county, state)
	With hospitals for clinical information OR web-only access
	With hospitals for demographic and insurance information
	With internal disease registries for case management
	With other clinics for clinical information
	With pharmacies or pharmacy clearinghouses (e.g. SureScripts)
	With reference laboratories
	With the Centers for Disease Control
	Ability to transmit standardized format (e.g. CCD, CCR) or other standardized individual components of patient's electronic record
	Ability to update the patient's EHR where there is a community-based HIE
**Domain: Patient use**	A patient portal allowing the patient to see personal health information, pay bills, request a schedule, request an appointment, etc.
	Email communications with physicians or nurses
	Patient Health Record
	Patient specific medical education content

Abbreviations: BMI=Body Mass Index, CCD=Continuity of Care Document, CCR=Continuity of Care Record, EMR=Electronic Medical Record, HIE=Health Information Exchange

© 2021 Shekelle PG et al. *JAMA Network Open.*

eTable 2. Performance Measures Publicly Reported at the Clinic Level in 3 States

Minnesota	
**Domain**	**Measure**
Screening	Colorectal Cancer Screening
Asthma	Adults
	Children
Depression	Depression - PHQ-9 Follow-up at 12 Months
	Depression - Use of the PHQ-9
	Depression Remission at 12 Months
	Depression Remission at Six Months
	Depression Response at 12 Months
	Depression Response at Six Months
	Depression: Follow Up (6 Months)
Mental Health	Mental Health Screening: Teens<br>Adolescent Measure by Clinic
Diabetes	All-or-none composite of care
Vascular Disease	All-or-none composite of care

Washington	
**Domain**	**Measure**
Antidepressants	Antidepressant medication - 12 weeks (measured in 2014-16)<br>Staying on antidepressant medication – 12 weeks (measured in 2017)
	Antidepressant medication - 6 months (measured in 2014-16)<br>Staying on antidepressant medication - 6 months (measured in 2017)
Child/adolescent well childcare	Adolescent well-care visits
	Well-child visits (ages 3-6 years)
Diabetes	Adherence for diabetes medications (measured in 2014-16)<br>Taking diabetes medications as directed (measured in 2017)
	Blood sugar (HBA1c) test (measured in 2014-16)<br>Blood sugar (HbA1c) testing for people with diabetes (measured in 2017)
	Diabetes Eye Exam (measured in 2014-16)<br>Eye exam for people with diabetes (measured in 2017)

© 2021 Shekelle PG et al. *JAMA Network Open.*

	Diabetes Kidney Disease Screening (measured in 2014-16)<br>Kidney disease screening for people with diabetes (measured in 2017)
Sore throats/colds/bronchitis	Appropriate testing for children with pharyngitis (measured in 2014-16)<br>Appropriate testing for children with sore throat (measured in 2017)
	Avoidance of antibiotics for common cold (measured in 2014-16)<br>Avoiding antibiotics for children with upper respiratory infection (measured in 2017)
	Avoidance of Antibiotic Treatment in Adults with Acute Bronchitis (measured in 2014-16)<br>Avoiding antibiotics for adults with acute bronchitis (measured in 2017)
CVD	Adherence for cholesterol medications – statins (measured in 2014-16)<br>Taking cholesterol-lowering medications as directed (measured in 2017)
	Adherence for hypertension medications - RAS antagonists (measured in 2014-16)<br>Taking hypertension medications as directed (measured in 2017)
	Cholesterol-lowering drugs – statins (measured in 2014-16)<br>Cholesterol-lowering medication generic prescriptions (measured in 2017)
Generic medication management	ADHD medication generic prescriptions
	Antidepressant medication generic prescriptions
	Generic Prescription Drugs - ACE inhibitors or angiotensin II receptor blockers – ARBs (measured in 2014-16)<br>High-blood pressure medication generic prescriptions (measured in 2017)
Cancer screening	Breast cancer screening
	Cervical cancer screening
	Colon cancer screening
Antacid Medication	Antacid medication - proton pump inhibitors (measured in 2014-16)<br>Stomach acid medication generic prescriptions (measured in 2017)
Asthma	Asthma - Use of Appropriate Medication (measured in 2014)<br>Managing medications for people with asthma (measured in 2015-17)
Back pain	Avoidance of x-ray, MRI and CT scan for low back pain (measured in 2014-16)<br>Avoiding X-ray, MRI and CT scan for low-back pain (measured in 2017)
STD screening	Chlamydia screening
Medication safety	Medication safety - monitoring patients on hypertension medications (measured in 2014-16)<br>Monitoring patients on high-blood pressure medications (measured in 2017)
**Wisconsin**	
**Domain**	**Measure**

© 2021 Shekelle PG et al. *JAMA Network Open.*

Vaccinations	Adults with Pneumococcal Vaccinations
Screening/treatment for tobacco	Tobacco User Receiving Tobacco Cessation Advice
Cancer screening	Breast Cancer Screening
	Cervical Cancer Screening
	Colorectal Cancer Screening
High blood pressure	Controlling High Blood Pressure: Blood Pressure Control
Diabetes	Diabetes: All-or-None Outcome Measure (Optimal Control)
	Diabetes: All-or-None Process Measure (Optimal Testing)
	Diabetes: Blood Pressure Control
	Diabetes: Blood Sugar (A1c) Control
	Diabetes: Blood Sugar (A1c) Testing
	Diabetes: Kidney Function Monitored
	Diabetes: Most Recent Tobacco Status is Tobacco-Free
	Diabetes: Statin Use Unless Contraindicated
	Diabetes: Daily Aspirin or Other Antiplatelet for Diabetes Patients with Ischemic Vascular Disease (IVD) Unless Contraindicated
Ischemic vascular disease	Ischemic Vascular Disease: All-or-None Outcome Measure (Optimal Control)
	Ischemic Vascular Disease: Blood Pressure Control
	Ischemic Vascular Disease: Daily Aspirin or Other Antiplatelet Therapy Unless Contraindicated
	Ischemic Vascular Disease: Most Recent Tobacco Status is Tobacco-Free
	Ischemic Vascular Disease: Statin Use Unless Contraindicated
Osteoporosis	Screening for Osteoporosis

Abbreviations: ACE inhibitors= Angiotensin converting enzyme inhibitors, ADHD=Attention Deficit Hyperactivity Disorder, CT=Computerized Tomography, CKD=Chronic Kidney Disease, eGFR= Estimated glomerular filtration rate, HBA1c=Hemoglobin A1c, LDL= Low-Density Lipoproteins, MRI=Magnetic Resonance Imaging, PHQ-9=Patient Health Questionnaire-9, RAS=Renin-angiotensin-system-acting agents

© 2021 Shekelle PG et al. *JAMA Network Open.*

eTable 3. Clinical Quality Composite Scores of Matched and Unmatched Ambulatory Care Sites in Cross-Sectional Analysis

Year	State	Match	# of Sites	Minimum	Mean	Maximum	Standard Deviation (SD)	*P* value (match v. non-match)
2014	MN	No	220	-2.86	-0.19	0.96	0.62	**< 0.001**
		Yes	327	-1.85	0.10	1.26	0.51	
2015		No	258	-3.14	-0.15	1.04	0.65	**< 0.001**
		Yes	309	-1.48	0.13	1.05	0.49	
2016		No	229	-2.88	-0.17	1.08	0.64	**< 0.001**
		Yes	356	-1.90	0.15	1.21	0.48	
2017		No	232	-2.36	-0.14	0.97	0.64	**< 0.001**
		Yes	323	-1.86	0.10	0.96	0.41	
2014	WA	No	277	-3.40	-0.58	2.16	0.94	**< 0.001**
		Yes	219	-3.50	-0.26	2.11	0.85	
2015		No	334	-2.58	-0.21	1.94	0.77	**< 0.001**
		Yes	289	-2.57	0.06	2.02	0.72	
2016		No	311	-2.28	0.03	2.19	0.66	**0.001**
		Yes	276	-1.75	0.20	2.28	0.63	
2017		No	453	-2.73	0.10	1.64	0.62	**0.003**
		Yes	419	-4.00	0.23	1.68	0.62	
2014	WI	No	81	-5.67	-0.26	0.78	1.02	0.205
		Yes	207	-2.85	-0.10	0.91	0.55	
2015		No	88	-2.24	-0.01	1.27	0.76	0.868
		Yes	270	-3.12	0.01	0.97	0.59	
2016		No	121	-2.08	0.18	1.61	0.64	0.936
		Yes	259	-2.93	0.17	1.19	0.58	
2017		No	107	-2.38	0.33	1.32	0.53	0.142
		Yes	259	-2.05	0.23	1.18	0.56	

Abbreviations: MN=Minnesota, WA=Washington, WI=Wisconsin

© 2021 Shekelle PG et al. *JAMA Network Open.*

eTable 4. Characteristics of Matched and Unmatched Ambulatory Care Sites in Cross-Sectional Analysis

Year	State	Match	# of Sites	# of Health Systems	Median # Clinics/ Health System (25%, 75%)	# Primary Care Clinics (%)	# Clinics in Multi-Hospital Systems (%)	# of Clinics reporting # of physicians in clinic	Median # of physicians per clinic (25%, 75%)
2014	MN	No	468	56	3 (2, 9)	160 (34%)	350 (75%)	451	2 (1, 6)
2014	MN	Yes	379	46	2 (1, 6)	208 (55%)	316 (83%)	374	6 (2, 12)
2015	MN	No	561	58	3 (2, 9)	187 (33%)	443 (79%)	530	3 (1, 7)
2015	MN	Yes	358	42	2 (1, 7)	201 (56%)	293 (82%)	355	6 (2, 12)
2016	MN	No	554	56	3 (2, 11)	161 (29%)	414 (75%)	513	3 (1, 7)
2016	MN	Yes	417	43	4 (1, 9)	233 (56%)	334 (80%)	409	6 (2, 11)
2017	MN	No	610	54	3 (2, 10)	188 (31%)	467 (77%)	560	3 (1, 8)
2017	MN	Yes	377	41	3 (1, 7)	209 (55%)	304 (81%)	373	6 (2, 11)
2014	WA	No	747	59	6 (2, 13)	236 (32%)	474 (63%)	695	2 (1, 4)
2014	WA	Yes	186	26	3 (2, 12)	115 (62%)	120 (65%)	175	6 (3, 10)
2015	WA	No	754	55	5 (2, 13)	200 (27%)	571 (76%)	716	2 (1, 4)
2015	WA	Yes	248	42	2 (1, 7)	144 (58%)	177 (71%)	239	5 (3, 9)
2016	WA	No	828	55	4 (2, 13)	205 (25%)	649 (78%)	746	2 (1, 4)
2016	WA	Yes	245	39	2 (1, 6)	147 (60%)	181 (74%)	229	5 (3, 9)
2017	WA	No	764	54	4 (2, 14)	197 (26%)	585 (77%)	678	2 (1, 4)
2017	WA	Yes	341	46	1.5 (1, 7)	161 (47%)	258 (76%)	316	5 (2, 9)
2014	WI	No	986	62	5.5 (2, 21)	341 (35%)	737 (75%)	922	2 (1, 6)
2014	WI	Yes	204	18	10.5 (4, 18)	128 (63%)	169 (83%)	197	5 (3, 9)
2015	WI	No	1094	59	7 (2, 21)	372 (34%)	888 (81%)	1056	2 (1, 5)
2015	WI	Yes	264	19	13 (7, 19)	160 (61%)	236 (89%)	252	6 (3, 11)
2016	WI	No	1078	58	6.5 (2, 20)	358 (33%)	875 (81%)	1037	2 (1, 5)
2016	WI	Yes	254	18	11.5 (4, 19)	154 (61%)	226 (89%)	243	5 (3, 11)
2017	WI	No	1174	60	6.5 (2, 19)	381 (32%)	991 (84%)	1120	2 (1, 5)
2017	WI	Yes	254	17	13 (6, 19)	155 (61%)	248 (98%)	243	6 (3, 11)

Abbreviations: MN=Minnesota, WA=Washington, WI=Wisconsin

eTable 5. Longitudinal Analysis: Clinic Capabilities

Longitudinal Progression	Number of Clinics	Mean Composite Difference	2017 Mean Composite
Static: Not Functional/Underuser	180	0.44	0.10
Static: Superuser	339	0.12	0.33
Non-identifiable	22	0.31	0.39
Not Functional to Underuser/Neither	19	0.75	0.35
Underuser to Neither	14	0.57	0.32
Neither to Superuser	48	0.46	0.25
Underuser to Superuser	24	0.35	0.27

© 2021 Shekelle PG et al. *JAMA Network Open.*

eFigure 1. Matching of Clinics in Washington, Wisconsin, and Minnesota, 2014 Cross-Sectional Analysis

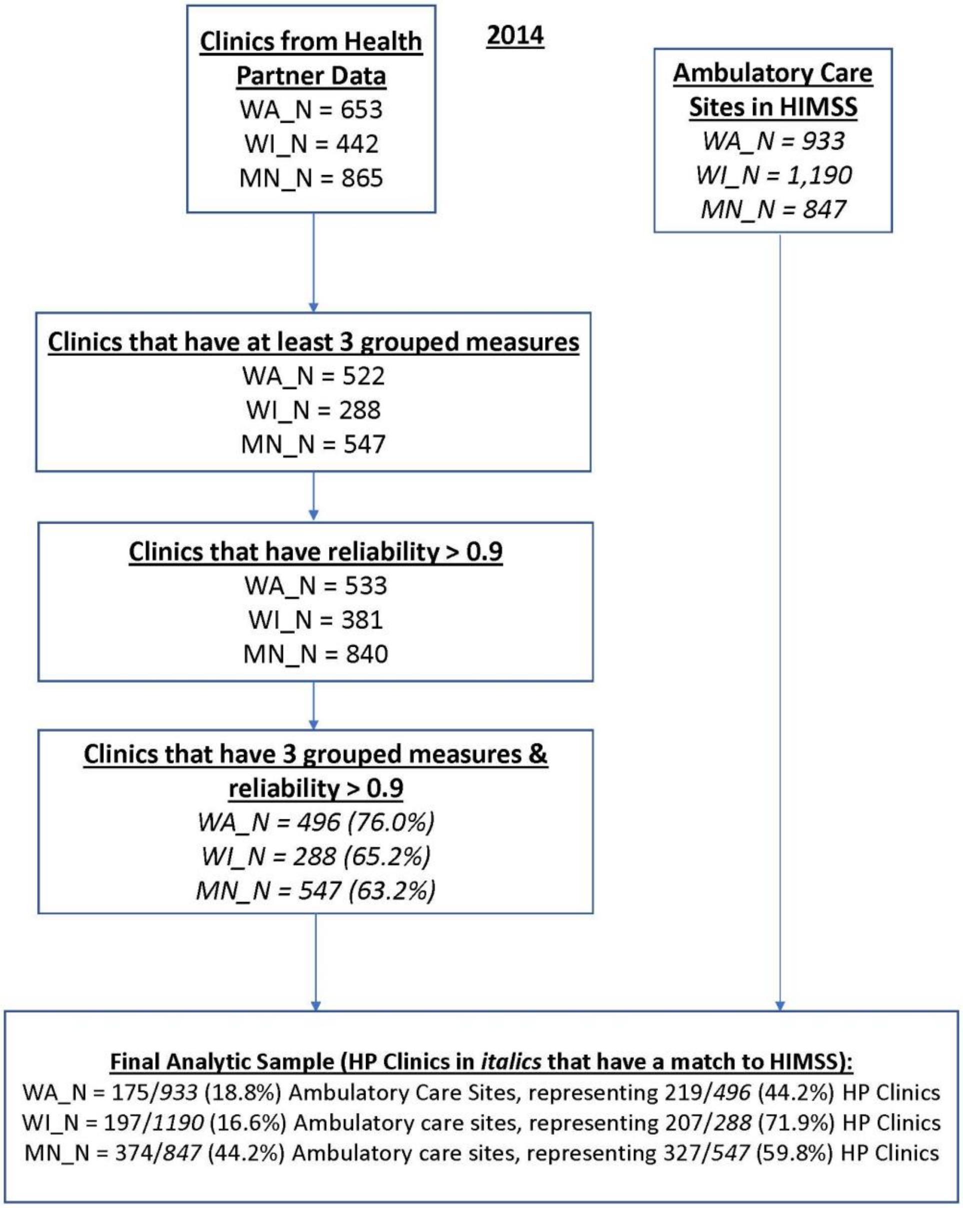

Abbreviations: HIMSS= Healthcare Information and Management Systems Society, HP=Health Partners, MN=Minnesota, WA=Washington, WI=Wisconsin

© 2021 Shekelle PG et al. *JAMA Network Open.*

eFigure 2. Matching of Clinics in Washington, Wisconsin, and Minnesota, 2015 Cross Sectional Analysis

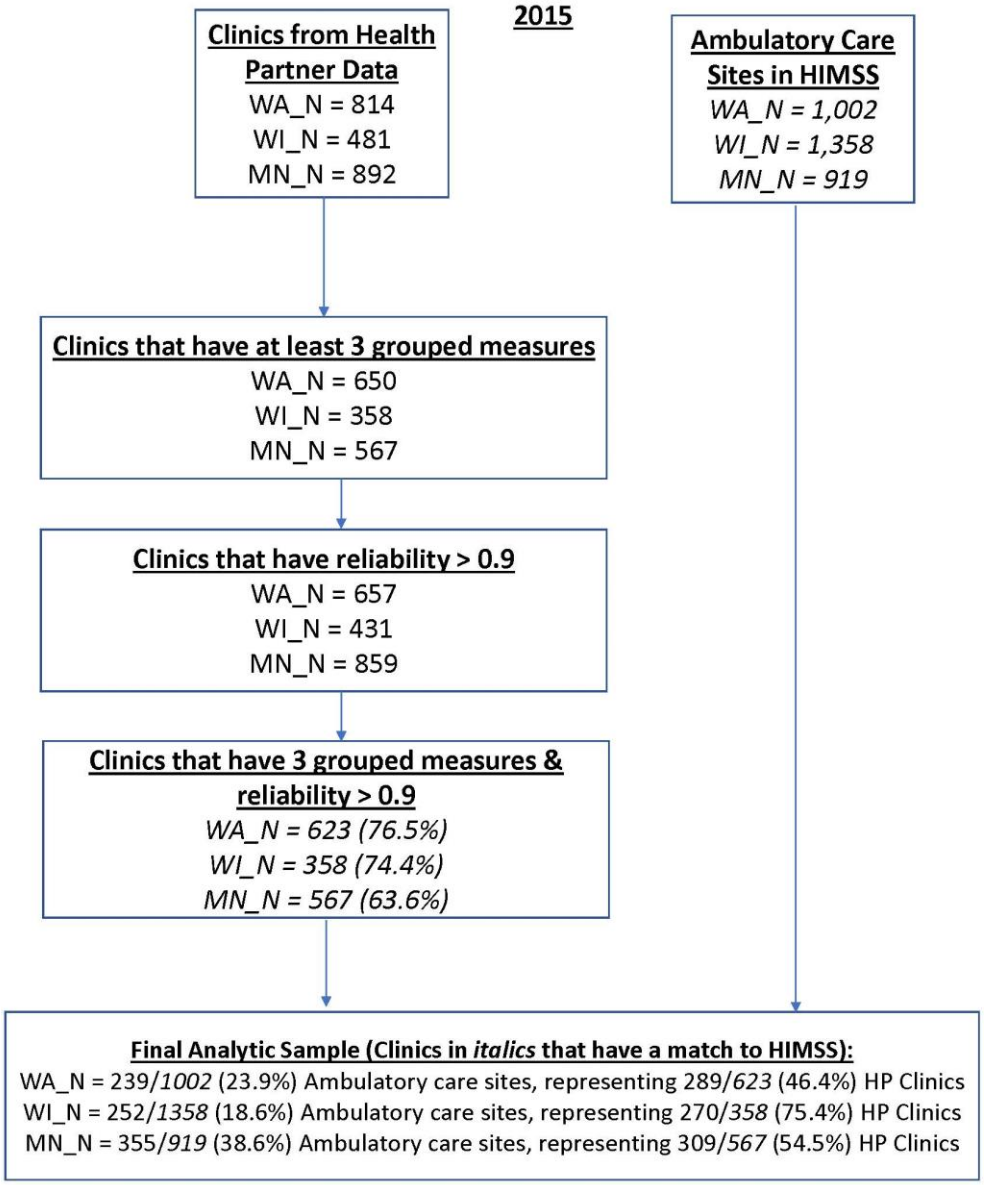

Abbreviations: HIMSS= Healthcare Information and Management Systems Society, HP=Health Partners, MN=Minnesota, WA=Washington, WI=Wisconsin

© 2021 Shekelle PG et al. *JAMA Network Open.*

eFigure 3. Matching of Clinics in Washington, Wisconsin, and Minnesota, 2016 Cross Sectional Analysis

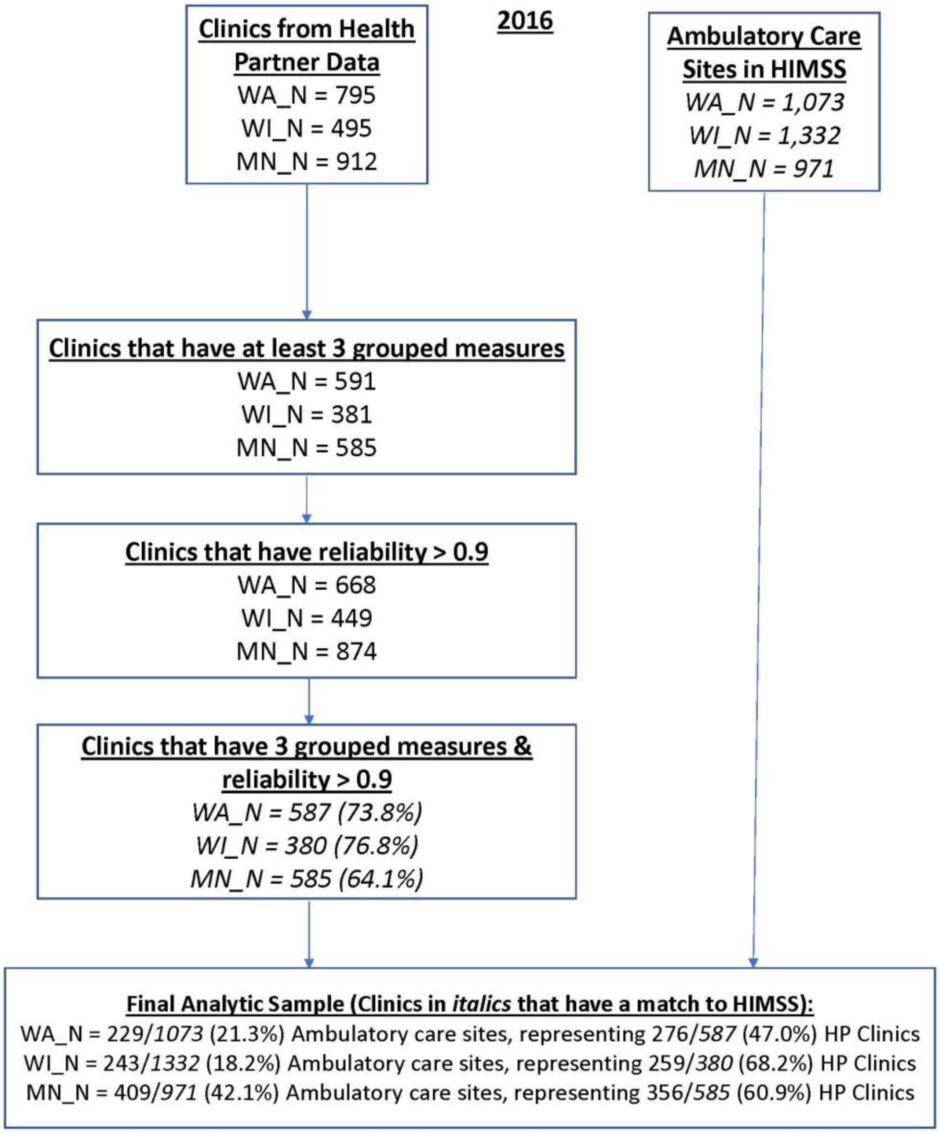

Abbreviations: HIMSS=Healthcare Information and Management Systems Society, HP=Health Partners, MN=Minnesota, WA=Washington, WI=Wisconsin

© 2021 Shekelle PG et al. *JAMA Network Open.*

eFigure 4. Matching of Clinics in Washington, Wisconsin, and Minnesota, 2017 Cross Sectional Analysis

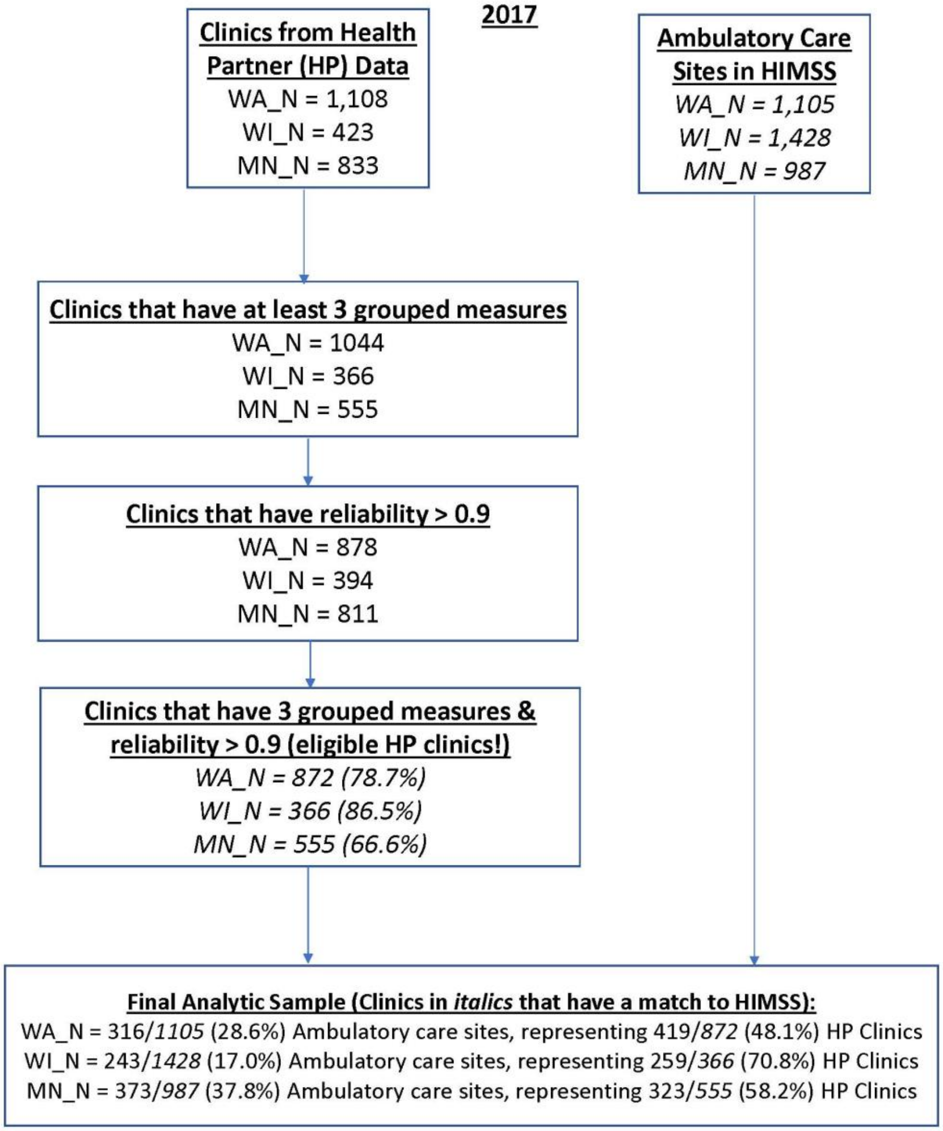

Abbreviations: HIMSS=Healthcare Information and Management Systems Society, HP=Health Partners, MN=Minnesota, WA=Washington, WI=Wisconsin

© 2021 Shekelle PG et al. *JAMA Network Open.*

eFigure 5. Matching of Clinics in Washington, Wisconsin, and Minnesota, Longitudinal Analysis, 2014-2017

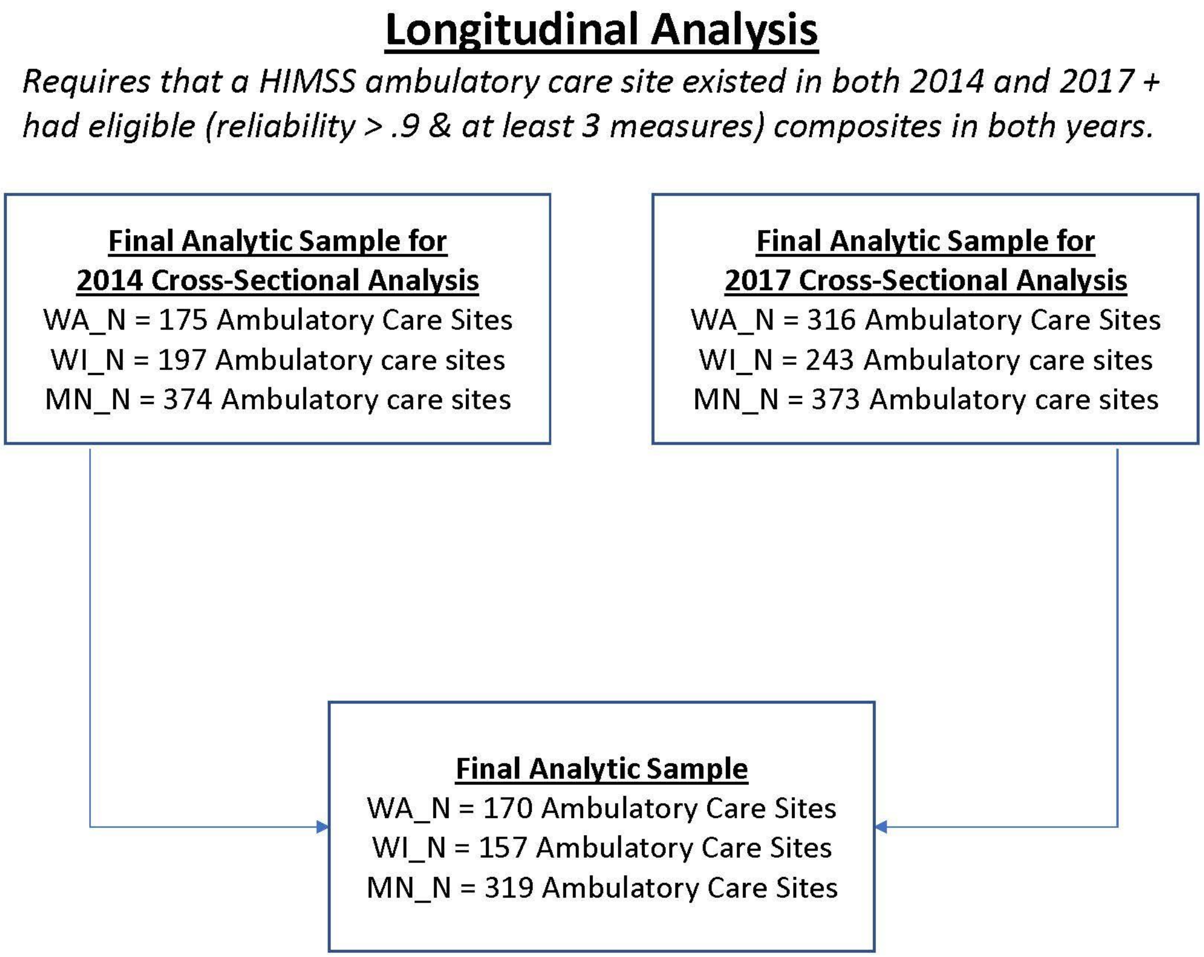

Abbreviations: MN=Minnesota, WA=Washington, WI=Wisconsin

© 2021 Shekelle PG et al. *JAMA Network Open.*
